# A Novel Nine Apoptosis-Related Genes Signature Predicting Overall Survival for Kidney Renal Clear Cell Carcinoma and its Associations with Immune Infiltration

**DOI:** 10.3389/fmolb.2021.567730

**Published:** 2021-03-04

**Authors:** Yi Wang, Yinhao Chen, Bingye Zhu, Limin Ma, Qianwei Xing

**Affiliations:** Department of Urology, Affiliated Hospital of Nantong University, Nantong, China

**Keywords:** clear cell renal cell carcinoma, apoptosis-related genes, overall survival, prognosis, signature

## Abstract

**Background:** This study was designed to establish a sensitive prognostic model based on apoptosis-related genes to predict overall survival (OS) in patients with clear cell renal cell carcinoma (ccRCC).

**Methods:** Obtaining the expression of apoptosis-related genes and associated clinical parameters from online datasets (The Cancer Genome Atlas, TCGA), their biological function analyses were performed through differently expressed genes. By means of LASSO, unadjusted and adjusted Cox regression analyses, this predictive signature was constructed and validated by internal and external databases (both TCGA and ArrayExpress).

**Results:** A total of nine apoptosis-related genes (SLC27A2, TNFAIP2, IFI44, CSF2, IL4, MDK, DOCK8, WNT5A, APP) were ultimately screened as associated hub genes and utilized to construct a prognosis model. Then our constructed riskScore model significantly passed the validation in both the internal and external datasets of OS (all *p* < 0.05) and verified their expressions by qRT-PCR. Moreover, we conducted the Receiver Operating Characteristic (ROC), finding the area under the ROC curves (AUCs) were all above 0.70 which indicated that riskScore was a stable independent prognostic factor (*p* < 0.05). Furthermore, prognostic nomograms were established to figure out the relationship between 1-, 3- and 5-year OS and individual parameters for ccRCC patients. Additionally, survival analyses indicated that our riskScore worked well in predicting OS in subgroups of age, gender, grade, stage, T, M, N0, White (all *p* < 0.05), except for African, Asian and N1 (*p* > 0.05). We also explored its association with immune infiltration and applied cMap database to seek out highly correlated small molecule drugs.

**Conclusion:** Our study successfully constructed a prognostic model containing nine hub apoptosis-related genes for ccRCC, helping clinicians predict patients’ OS and making the prognostic assessment more standardized. Future prospective studies are required to validate our findings.

## Introduction

Renal cancer, one of the most familiar malignant urological cancers in the world, attributes to 73,820 new cases and 14,770 deaths every year in the United States (Siegel et al., 2019). Therein, renal cell cancer (RCC) consists of 90% of renal carcinoma and it has been reported that almost 400,000 cases were diagnosed in 2018 (Bray et al., 2018). The most common type is clear cell renal cell carcinoma (ccRCC) which causes a low risk of cancer-related death when diagnosed (Joseph et al., 2014). However, almost one third of these newly diagnosed patients had metastases and a poor prognosis (Dagher et al., 2017). Although targeted therapeutics and immune checkpoint inhibitors have changed the landscape of treatment for ccRCC, most patients still did not experience significant clinical benefits (Orlandella et al., 2020). Accordingly, it was critical to discover more effective methods for early screening and diagnosis of ccRCC by further understanding its molecular mechanism and biological process. Thus, we can improve the treatment effect and life quality of these ccRCC patients.

Programmed cell death is a highly conserved and strictly regulated cellular program. Apoptosis occurs in various physiological conditions and pathological states (Lockshin and Zakeri, 2007; Fuchs and Steller, 2011). If excessive or insufficient apoptosis, or occurrence at the wrong time or place, it may lead to pathological development, such as stroke and cancer (Barinaga, 1998; Li et al., 2010). Increasing apoptosis and decreasing cell death could increase the occurrence of cancer and promote the progress of cancer (Fulda, 2009). As previously reported, there were many apoptosis-related genes, including the caspase family, the tumor suppressor gene P53, the oncogene C-myc, the IAPs, FLIPs and the Bcl-2 family (Packham and Cleveland, 1995; Bellamy, 1997; Mohamed et al., 2017; Edlich, 2018; Humphreys et al., 2018). Furthermore, they are involved in abnormal gene expression or genomic changes, leading to changes in apoptosis pathways that enable cancer cells to escape apoptosis (Hanahan and Weinberg, 2000). For instance, PAR4 can induce apoptosis of cancer cells without damaging normal cells (El-Guendy and Rangnekar, 2003). Survivin expression was increased in gastric cancer, and RUNX3 promoted apoptosis by inhibiting survivin expression (Liu et al., 2014). Notch signaling played important roles in the occurrence of tumor and therapeutic resistance of NSCLC cells via the promotion of proliferation or the inhibition of apoptosis (Zou et al., 2018).

In past decades, numerous diagnostic biomarkers associated with ccRCC have been found (Gong et al., 2006; Ricketts et al., 2012; Lu et al., 2016; Yadav et al., 2017), but it could rarely conduct accurate and sensitive early-stage detection, thus making the ccRCC patients lose the chance to be cured early. Moreover, the implementation of cancer precision medicine requires biomarkers or signatures for predicting prognosis and therapeutic benefits. Hence, there remains an urgent need for improving the performance of biomarkers or signatures. In this study, we concentrated our work on constructing a prognostic signature related to apoptosis-related genes to calculate risk level and predict survival time in ccRCC patients. We validated the prognostic model via internal and external aspects. Our results were anticipated for creating novel standard of diagnosis and developing therapeutic targets, helping clinician predict ccRCC patients’ OS more effectively and accurately.

## Materials and Methods

### Data Acquisition and Processing

The RNA-sequencing datasets and related clinical data of 72 normal renal tissue samples and 539 ccRCC samples were acquired from The Cancer Genome Atlas database (TCGA, https://portal.gdc.cancer.gov). The census of human apoptosis-related genes was obtained from GSEA (https://www.gsea-msigdb.org/gsea/index.jsp) and these gene sets are displayed in the Supplementary Table S1. To ensure a unified standard, the RNA-seq profiles were transformed using the formula log2 (x+1) and normalized. To obtain the differently-expressed genes in the ccRCC tissue, we utilized “limma” package on account of false discovery rate (FDR) < 0.05 and |log2 fold change (FC)| ≥ 1. Besides, we downloaded 99 samples from ArrayExpress datasets (E-MTAB-1980, https://www.ebi.ac.uk/arrayexpress/) as the external validation.

### GO and KEGG Functional Enrichment Analyses

Based on differently-expressed apoptosis-related genes, the gene ontology (GO) enrichment involving molecular function (MF), cellular components (CC), and biologic process (BP) was conducted and the kyoto encyclopedia of genes and genomes (KEGG) pathway analysis was also performed. GO and KEGG pathway analysis were conducted to annotate genes and identify biological attributes for differentially expressed apoptosis-related genes. Both FDRs and *p* values were significant when they were less than 0.05.

### PPI Network Construction and Module Screening

The differently-expressed apoptosis-related genes were uploaded to the STRING database (http://www.string-db.org/) to further acquire protein-protein interaction relationship. Then we performed the next analysis by Cytoscape 3.7.0 software to construct the PPI network. The key genes and modules were selected in PPI network with the plug-in of Molecular Complex Detection (MCODE) on the condition of both MCODE scores and the number of nodes are more than 5 (Szklarczyk et al., 2019). All *p* ≤ 0.05 were considered as statistical significance.

### Prognostic Model Construction and Analyses

We firstly performed survival R package to conduct unadjusted Cox regression analysis on all apoptosis-related genes. Then the least absolute shrinkage and selection operator (LASSO) was executed to prevent the occurrence of overfitting and to further obtain the candidate genes. Afterwards, based on the previously acquired candidate genes, adjusted Cox proportional hazards regression was utilized to establish a calculation formula. Subsequently, a riskScore algorithm was constructed by above mentioned methods and displayed as follows:

$$\text{Risk score}=\sum_{\text{i}=1}^{\text{n}}\text{expi*}\beta \text{i}.$$

Therein, *β* means the coefficient value, and exp equals to the expression level.

### External and Internal Verification of the Prognostic Model

To evaluate the prognostic ability of our established model, we divided ccRCC patients into high- and low-risk groups based on the median risk score. A log-rank test was conducted to compare these two groups. In addition, we implemented a time-dependent ROC analysis and calculated the area under the ROC curve (AUC) by applying the SurvivalROC package to ccRCC patients. ArrayExpress datasets was set as the external validation. As for internal validation, the whole TCGA database was randomly divided into two groups for verification. In order to predict the likelihood of OS, we implemented the nomogram and calibration plots with rms R package. *p* < 0.05 was counted as statistical significance.

### Construction and Verification of the Prognostic Nomogram

According to prognostic clinical parameters and our established riskScore model, we constructed a nomogram to predict the possibilities of 1-, 3- and 5-year OS for ccRCC patients. ROC curves and their associated AUCs were applied to evaluate the sensitivity and accuracy of the prognostic nomogram. Further, the same methods were applied to the external ArrayExpress datasets to verify the outcomes.

### Validation of Apoptosis-Related Genes in ccRCC Tissues by Quantitative Real-Time PCR

To extract total RNA from the target tissue samples, after adding liquid nitrogen in a mortar to fully grind, we increased 1 ml Trizol reagent (Life technology, Grand Island, NY, United States) directly to lyse at room temperature for 15 min on a shaker. To evaluate expressing levels of mRNA, the first-strand cDNA was conducted using parts of total RNA applying the RevertAid First Strand cDNA Synthesis Kit (thermo scientific, Lithuania). Quantitative PCR was performed by Roche LightCycler^®^ 480 Real-Time PCR System with SYBR^®^ Green qPCR mix 2.0 kit for measurement. The primers applied in this study are obtained from TsingKe biological technology (Nanjing, China), including β-actin (forward 5′-ATG​ACT​TAG​TTG​CGT​TAC​ACC-3′, reverse 5′-GAC​TTC​CTG​TAA​CAA​CGC​ATC-3′); APP (forward 5′-TCT​CGT​TCC​TGA​CAA​GTG​CAA-3′, reverse 5′-GCA​AGT​TGG​TAC​TCT​TCT​CAC​TG-3′); CSF2 (forward 5′- AAT​GTT​TGA​CCT​CCA​GGA​GCC-3′, reverse 5′-GAG​GGC​AGT​GCT​GCT​TGT​AG-3′); DOCK8 (forward 5′-TAC​ATC​CGT​GAG​TGG​CTA​ATC​G-3′, reverse 5′- CGG​AAG​CGT​CTT​GTG​AAA​ATC​TT-3′); IFI44 (forward 5′-ATG​GCA​GTG​ACA​ACT​CGT​TTG-3′, reverse 5′-TCC​TGG​TAA​CTC​TCT​TCT​GCA​TA-3′); IL4 (forward 5′-CGG​CAA​CTT​TGT​CCA​CGG​A-3′, reverse 5′-TCT​GTT​ACG​GTC​AAC​TCG​GTG-3′); MDK (forward 5′-CGC​GGT​CGC​CAA​AAA​GAA​AG-3′, reverse 5′-TAC​TTG​CAG​TCG​GCT​CCA​AAC-3′); SLC27A2 (forward 5′- TAC​TCT​TGC​CTT​GCG​GAC​TAA-3′, reverse 5′-CCG​AAG​CAG​TTC​ACC​GAT​ATA​C-3′); TNFAIP2 (forward 5′-CTG​ACG​AAT​TAC​AGG​GCC​AAT-3′, reverse 5′- TGC​GTG​AAC​CTC​TTG​AAC​AGT-3′); WNT5A (forward 5′-CTG​GCA​GGA​CTT​TCT​CAA​GG-3′, reverse 5′-CCT​TCG​ATG​TCG​GAA​TTG​AT-3′). Tumor tissues and paired normal tissues were collected from six ccRCC patients from Affiliated Hospital of Nantong University and the present study was approved by the Institutional Research Ethics Committees of Affiliated Hospital of Nantong University. Besides, the relative mRNA level was calculated using 2^−ΔΔCt^ method.

### Estimation of Tumor-Infiltrating Immune Cells

We applied the “limma” package of R software to conduct analyses including the expression of TIICs in every sample and the inner relationship of these TIICs in ccRCC, showing by the heatmap and matrix. Moreover, the R package was performed to find whether there are high correlations with the risk model by applying the samples with high-and low-groups. After normalizing gene expression data with standard annotation files, we uploaded these data to the CIBERSORT web portal (Newman et al., 2015). Then we conducted the algorithm with LM22 gene signature and 1,000 permutations. The threshold above was set as the *p*-value less than 0.05.

### Identification of Potential Small Molecule Drugs

To efficiently seek out molecule drugs highly related to ccRCC, we applied a gene expression profiles database named connectivity map (cMap) (Lamb et al., 2006). Then we uploaded the differently expressed apoptosis-related genes to it and bioactive chemicals and potential connections were further explored. Scores of connectivity were set from −1 to 1 to evaluate the degree of closeness in the compound correlated to the query signature. Ultimately the positive score means the promotion of the drug, while negative scores could be considered as the function of depression by a drug. And the set threshold was *p* < 0.05, n ≥ 4 and |mean| > 0.4.

### Statistical Analysis

Statistical analysis was completed with the R software 3.6.3. To accomplish the comparison between low- and high-risk groups, Kaplan-Meier was conducted by applying the log-rank test. By means of LASSO, unadjusted and adjusted Cox regression analyses, we calculated the regression coefficient and the hazard ratio. The time-dependent Receiver Operating Characteristic (ROC) analysis and the area under the ROC curve (AUC) were respectively performed to assess the accuracy of the model and the nomogram. And we applied harrell’s index of concordance (C-index) to further evaluate the ability of the nomogram. *p* values < 0.05 were adhered in the whole statistical analyses.

## Results

### Identification of Differently Expressed Apoptosis-Related Genes in ccRCC Patients

**FIGURE 1:**
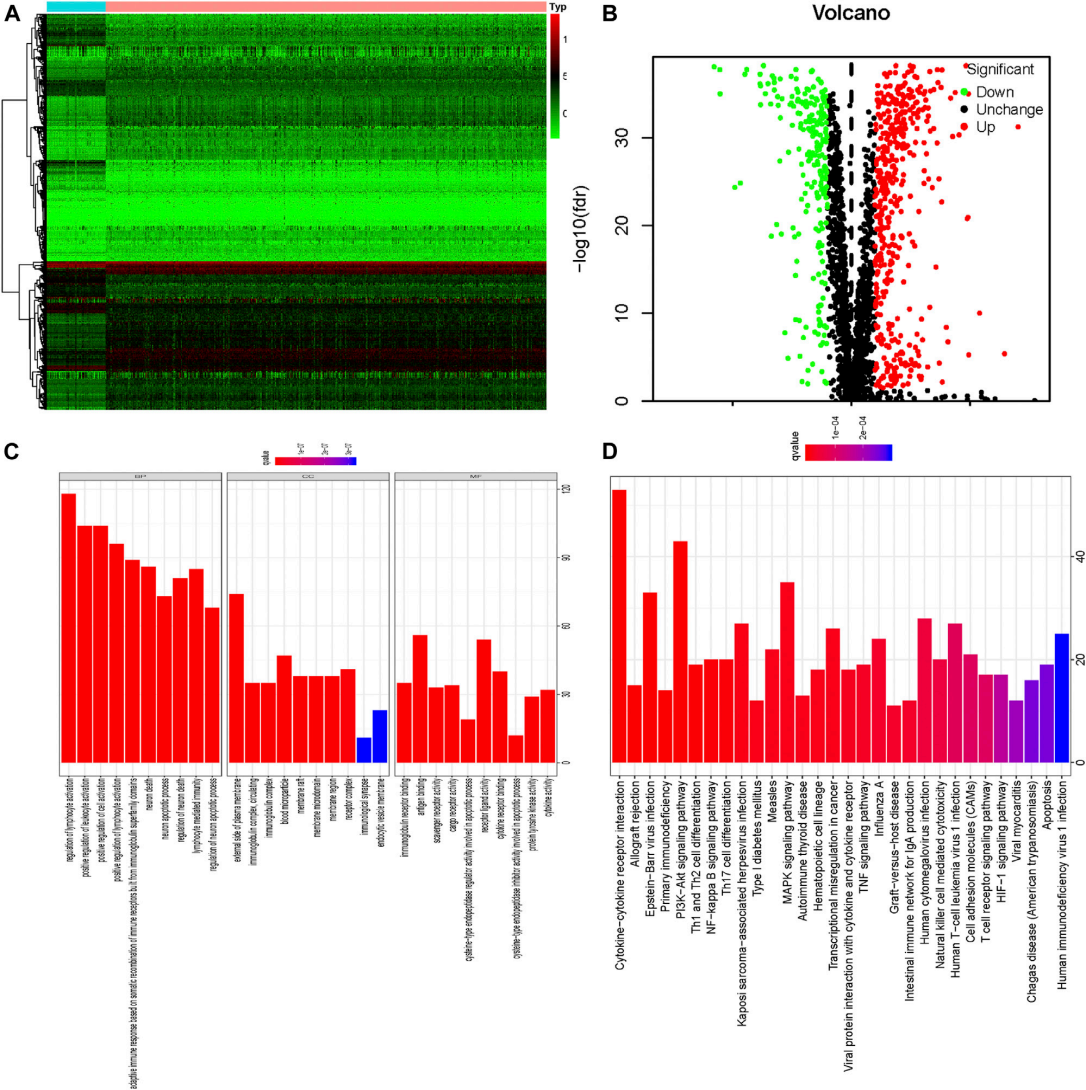
The 127 differentially expressed apoptosis-related genes identified in ccRCC; **(A)** Heatmap of these 127 differently expressed apoptosis-related genes; **(B)** Volcano plot of these 127 differently expressed apoptosis-related genes including 39 down-regulated and 88 up-regulated genes; Red nodes mean the up-regulated apoptosis-related genes, log2 (FC) > 1 and *p* < 0.05. Green nodes represent the down-regulated apoptosis-related genes, log2 (FC)< −1 and *p* < 0.05. **(C)** GO enrichment of 127 differently expressed apoptosis-related genes; **(D)** KEGG pathway analysis of 127 differently expressed apoptosis-related genes.

In this study, systematic analyses were conducted by several advanced computer technological methods to detect key roles and investigate prognostic values of apoptosis-related genes in ccRCC. The procedures of this study were illustrated in Supplementary Figure S1. The dataset of renal clear cell carcinoma was acquired from TCGA containing 539 tumor samples and 72 normal renal tissue samples. Then we conducted R software packages to cope with the data, thus finding the differently-expressed apoptosis-related genes. A total of 2,411 apoptosis-related genes were included in the above analysis, and 127 apoptosis-related genes met the accepted standard (FDR < 0.05, |log2 (FC)| > 1), consisting of 88 up-regulated and 39 down-regulated apoptosis-related genes. To show the differential expression of apoptosis-related genes, heat map and volcano plot were utilized respectively (Figures 1A,B).

### GO and KEGG Pathway Enrichment Analysis of the Differently Expressed Apoptosis-Related Genes

Our results indicated that differently expressed apoptosis-related genes were significantly enriched in the biological processes (BP) related to regulation of lymphocyte activation, positive regulation of leukocyte activation, positive regulation of cell activation, adaptive immune response based on somatic recombination of immune receptors built from immunoglobulin superfamily domains, neuron death, and neuron apoptotic processes. According to molecular function (MF), the differently expressed apoptosis-related genes were especially enriched in immunoglobulin receptor binding, antigen binding, scavenger receptor activity, cargo receptor activity, cysteine-type endopeptidase regulator activity involved in apoptotic process, and receptor ligand activity. We also found that differently-expressed apoptosis-related genes were enriched in the cellular component (CC) associated with all the components of the external plasma membrane that the genes were expressed in, immunoglobulin complex, blood microparticle, and membrane raft (Figure 1C; Supplementary Table S2). Additionally, results of KEGG pathway analysis indicated that apoptosis-related genes were mainly enriched in Cytokine-cytokine receptor interaction, Allograft rejection, Epstein-Barr virus infection, Primary immunodeficiency, PI3K-Akt signaling pathway, Th1 and Th2 cell differentiation, NF-kappa B signaling pathway and Th17 cell differentiation (Figure 1D, Supplementary Table S3).

### Protein-Protein Interaction Network Construction and Key Modules Selecting

**FIGURE 2:**
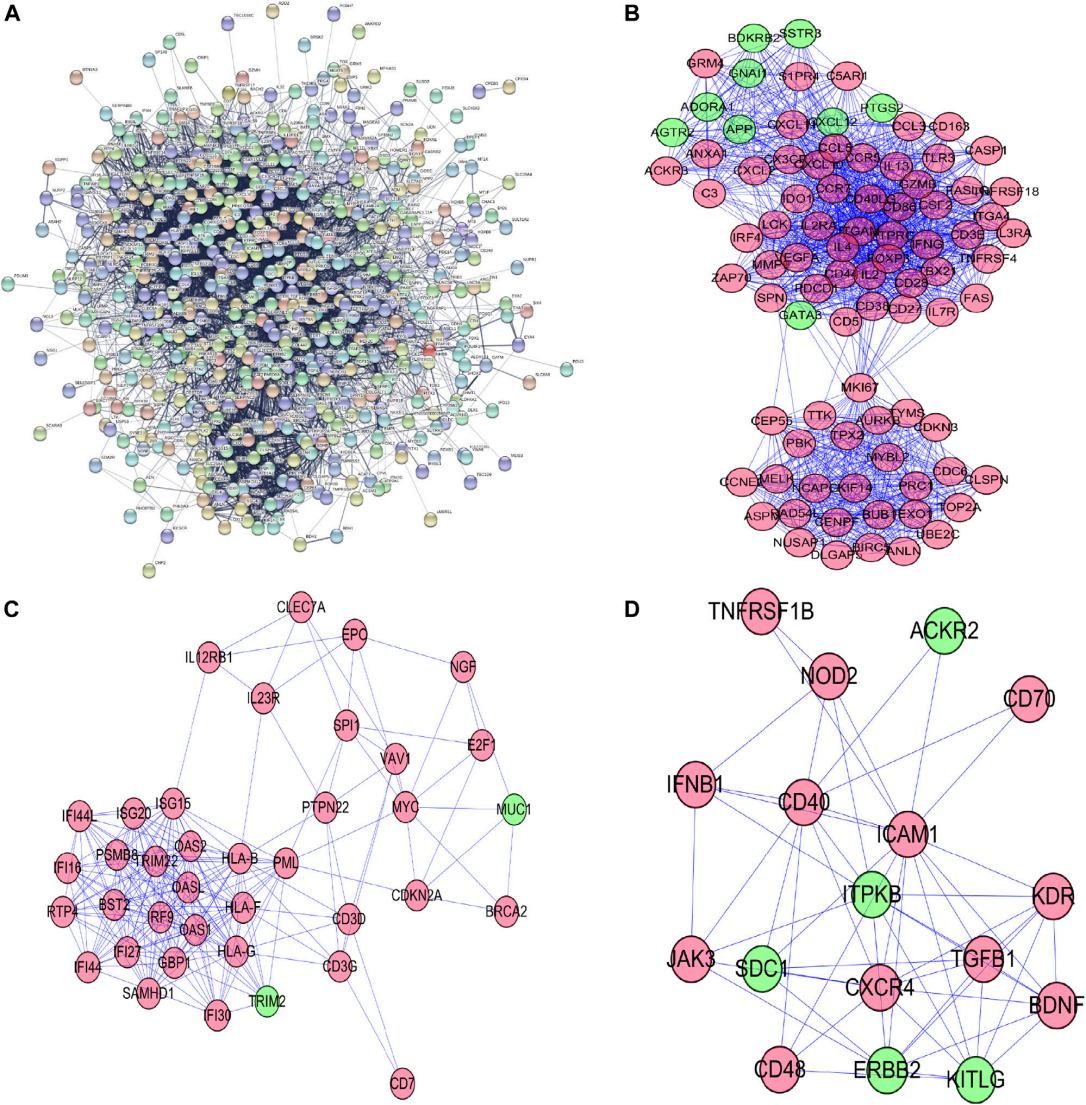
Protein–protein interaction network and its top 3 key modules analysis; **(A)** Protein–protein interaction network of differentially expressed apoptosis-related genes; **(B)** key module 1 from PPI network; **(C)** key module 2 from PPI network; **(D)** key module 3 from PPI network.

To further figure out the roles of differently-expressed apoptosis-related genes in ccRCC, we constructed a PPI network from the STRING database, consisting of 559 nodes and 6,096 edges (Figure 2A). To seek out possible key modules involved in the co-expression network, we utilized the MODE tool and finally screened the top 3 significant modules, by means of cytoscape software with the data from the STRING database (Figures 2B–D).

### Selection of Prognostic Apoptosis-Related Genes and Risk Score Model Construction

**FIGURE 3:**
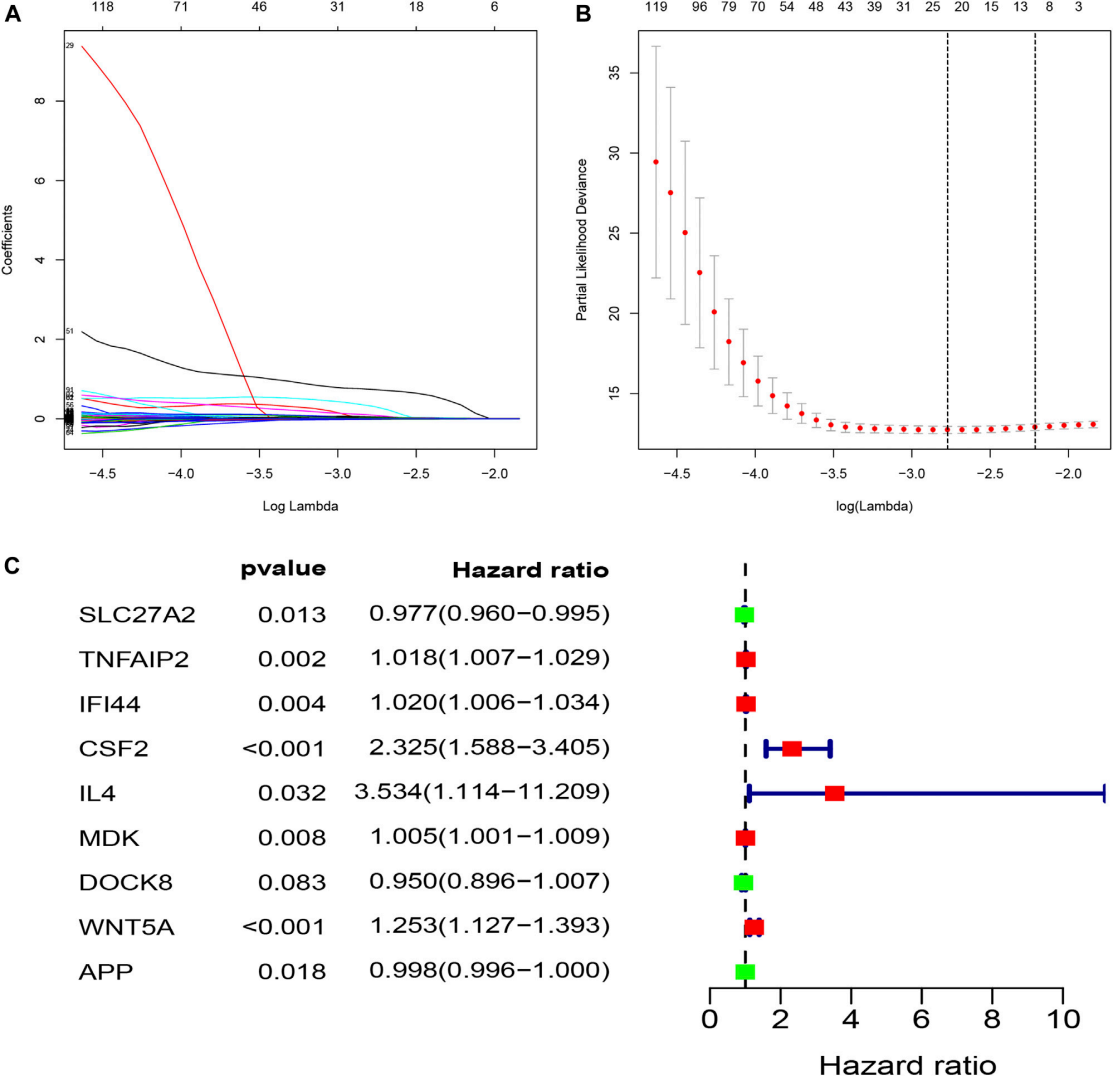
Construction of a prognostic model (riskScore) by means of LASSO and adjusted Cox regression analysis; **(A,B)** LASSO coefficients profiles of the prognostic apoptosis-related genes; The partial likelihood deviance plot presented the minimum number corresponds to the covariates. utilized for adjusted Cox regression analysis; **(C)** The forest plot of differently expressed apoptosis-related genes were identified to be significantly related to OS of ccRCC patients by adjusted Cox regression analysis. Green points represent negative correlations, whereas red points represent positive correlations.

To investigate the prognostic value of these apoptosis-related genes, we performed a unadjusted Cox regression analysis and obtained 276 prognostic apoptosis-related genes (Supplementary Table S4). We then conducted the least absolute shrinkage and selection operator (LASSO) method to prevent the occurrence of overfitting and obtained 21 critical genes closely relevant to OS (Figures 3A,B). These 21 prognostic apoptosis-related genes were further analyzed by adjusted Cox regression analysis and nine apoptosis-related genes were finally identified (Figure 3C; Table 1). Therefore, the score model composed of βi and the level of expression was constructed as follows:

**TABLE 1:** Nine prognostic hub apoptosis-related genes identified by adjusted Cox regression analysis.

Id	Coef	HR	HR.95L	HR.95H	*p* value
SLC27A2	−0.02303	0.977237	0.959638	0.995159	0.013017
TNFAIP2	0.017706	1.017864	1.00668	1.029171	0.001683
IFI44	0.019847	1.020045	1.006385	1.033892	0.003912
CSF2	0.843843	2.325286	1.587915	3.405066	1.45E-05
IL4	1.262371	3.533789	1.114081	11.20894	0.032082
MDK	0.005039	1.005052	1.001289	1.00883	0.008471
DOCK8	−0.05144	0.949859	0.896264	1.006658	0.082559
WNT5A	0.225475	1.252917	1.126575	1.393428	3.22E-05
APP	−0.00218	0.997825	0.99602	0.999632	0.01834

RiskScore = (−0.0230258171190983 * ExpSLC27A2) + (0.0177060929387359 * ExpTNFAIP2) + (0.0198471248627109 * ExpIFI44) + (0.843843107512985 * ExpCSF2) + (1.26237059569624 * ExpIL4) + (0.00503928604182937 * ExpMDK) + (−0.0514420718061081* ExpDOCK8) + (0.225474608019405 * ExpWNT5A) + (−0.00217783758241253 * ExpAPP)

### The External and Internal Validation of Prognosis-Related Genetic Risk Score Model

**FIGURE 4:**
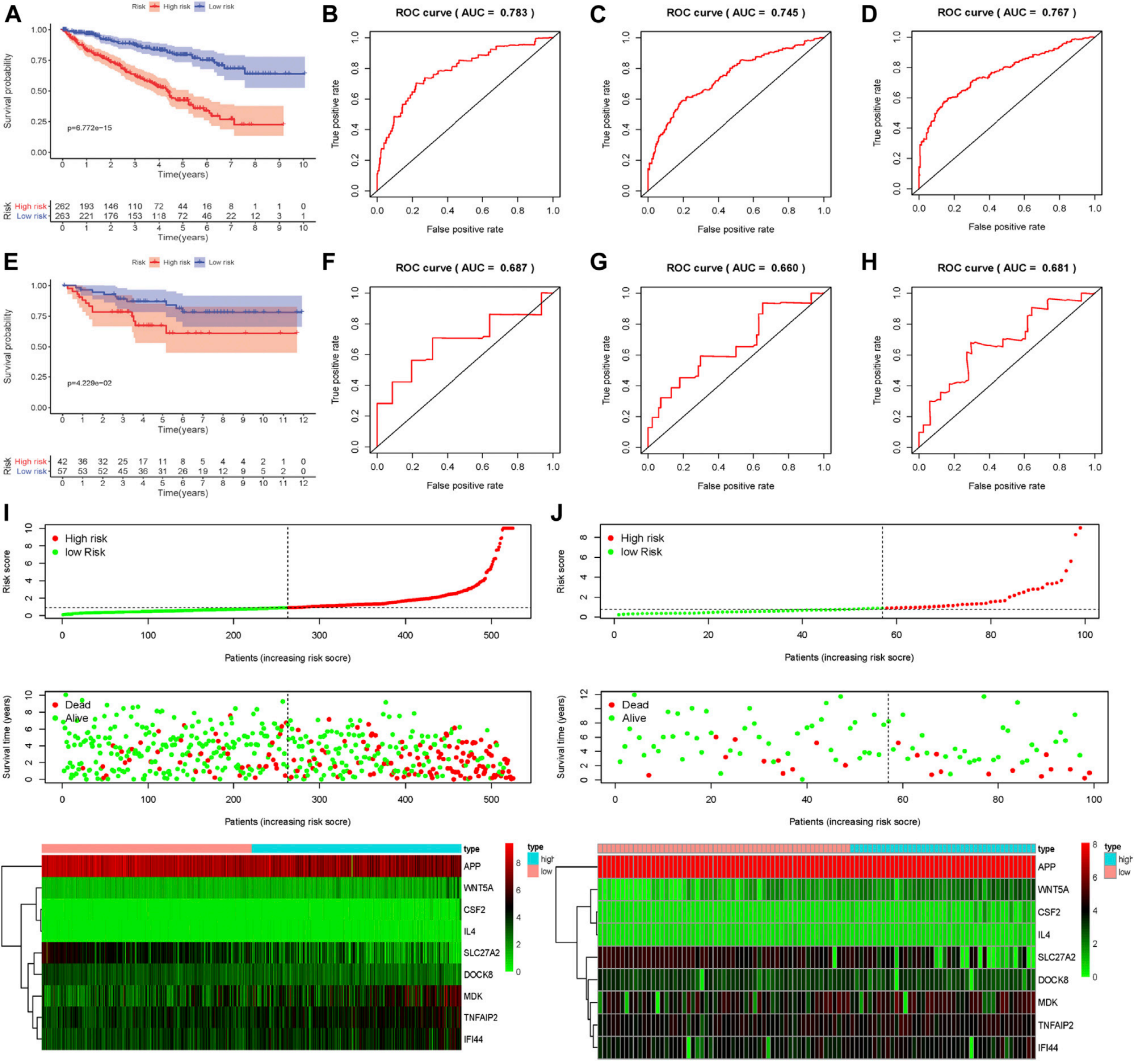
Evaluation and external verification of nine apoptosis-related genes established model; **(A)** Survival curves of OS from high- and low-risk groups of high- and low-risk divided by riskScore in the TCGA dataset; **(B–D)** 1-, 3- and 5-year ROC in the TCGA dataset; **(E)**Survival curves of OS from high- and low-risk groups of high- and low-risk divided by riskScore in the external validation dataset (ArrayExpress); **(F–H)** 1-, 3- and 5-year ROC in the ArrayExpress dataset; **(I**,**J)** The distribution of risk scores for samples, the survival status of patients and the expression heatmap of nine differently expressed genes in the TCGA dataset and the ArrayExpress dataset.

To assess the predictive ability and accuracy of the signature, we then conducted a survival analysis and performed verification in both TCGA and ArrayExpress databases. According to the median risk score of our established model, we divided the ccRCC patients into low- and high-risk subgroups. Results of the TCGA dataset indicated that patients in the low-risk subgroups had longer OS, compared with those in the high-risk subgroups (Figure 4A). To further evaluate the prognostic sensitivity of these nine apoptosis-related genes biomarker, we performed a time-dependent ROC analysis, thus finding that the AUCs of 1-year, 3-years and 5-year risk score model were 0.783, 0.745, 0.767, respectively (Figures 4B–D and Table 2), indicating a moderate diagnostic performance. Survival status of patients, expression heat map, and risk score of the signature were also shown in Figure 4I, finding that the higher the risk scores, the higher the patients in high-risk subgroups, and the higher the numbers of dead persons. Heatmap of these nine apoptosis-related genes between low- and high-risk subgroups was also displayed in this figure.

**TABLE 2:** External and internal verification datasets of 1-, 3- and 5-years ROC.

Datasets	1-year ROC	3-year ROC	5-year ROC
The TCGA dataset	0.783	0.745	0.767
The external ArrayExpress dataset	0.687	0.660	0.681
The internal validation dataset 1 (test 1)	0.734	0.737	0.770
The internal validation dataset 2 (test 2)	0.824	0.754	0.766

As for external verification, we applied the same formula to the ArrayExpress datasets (E-MTAB-1980) to evaluate whether the predictive model had similar prognostic value and accuracy in other ccRCC patient cohorts. By means of conducting survival analysis, we found that patients with low-risk scores also had a higher OS than those with high-risk scores in the ArrayExpress cohorts (Figure 4E). ROC curves and their associated AUCs were also performed and we found that the AUCs of 1-year, 3-years and 5-years risk score model were 0.687, 0.660, 0.681, separately (Figures 4F–H and Table 2). Figure 4J indicated that the higher the risk scores, the higher the patients in high-risk subgroups, and the higher the numbers of dead persons. Heatmap of these nine apoptosis-related genes between low- and high-risk subgroups was also displayed in this figure.

**FIGURE 5:**
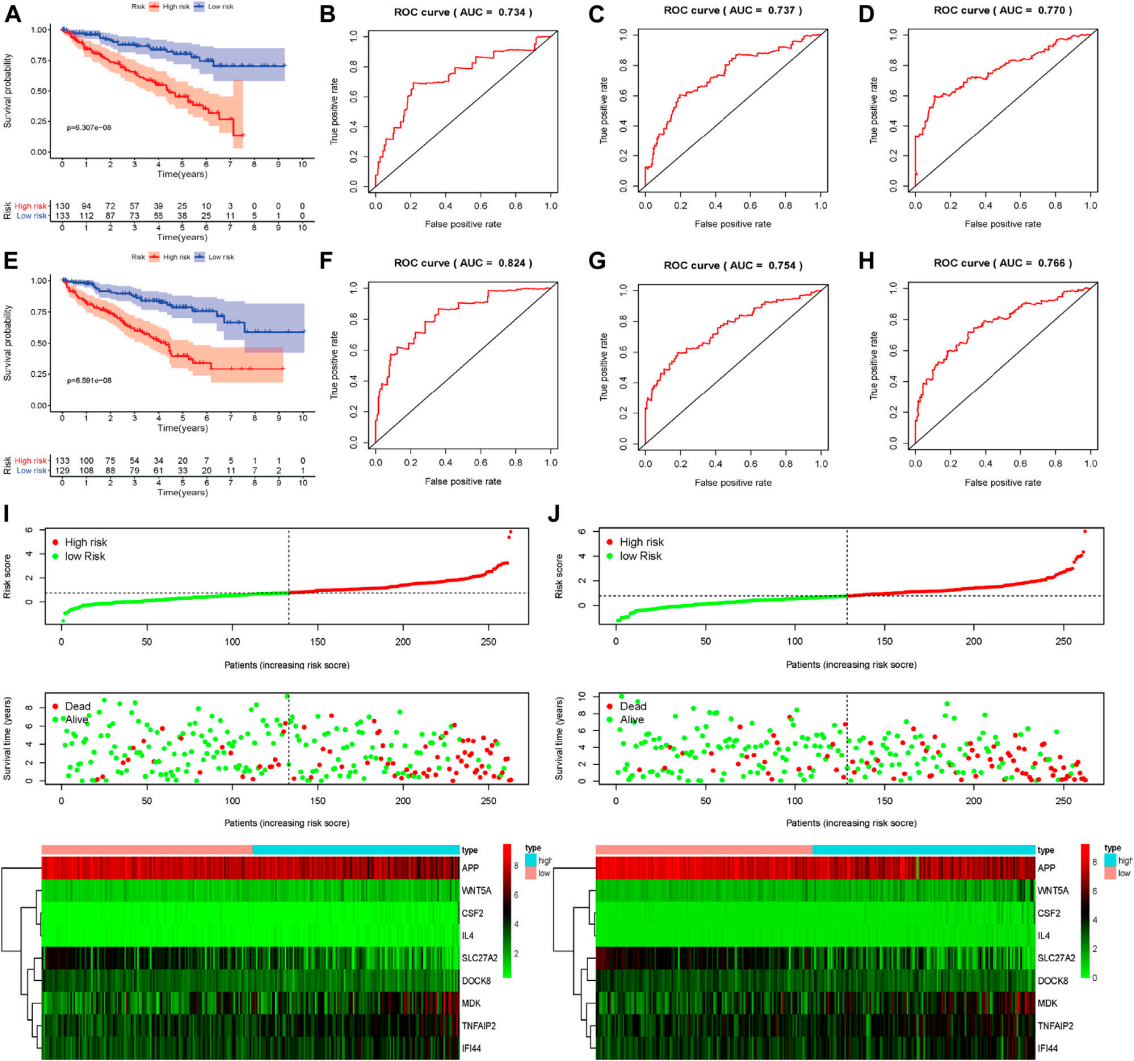
Internal verification of nine apoptosis-related genes signature; **(A)** Survival curves of OS from high- and low-risk groups of high- and low-risk divided by riskScore in the internal validation dataset 1 (test 1); **(B–D)** 1-, 3- and 5-year ROC in the internal validation dataset 1 (test 1); **(E)** Survival curves of OS from high- and low-risk groups of high- and low-risk divided by riskScore in the internal validation dataset 2 (test 2); **(F–H)** 1-, 3- and 5-year ROC in the internal validation dataset 2 (test 2); **(I**,**J)** The distribution of risk scores for samples, the survival status of patients and the expression heatmap of nine differently expressed genes the internal validation dataset 1 (test 1) and in the internal validation dataset 2 (test 2).

To conduct the internal verification, a total of 539 ccRCC samples in TCGA dataset were randomly divided into two groups including the internal validation dataset 1 (test 1) and the internal validation dataset 2 (test 2). Then we conducted the whole procedure of the internal verification of these two groups separately as the same as what we had performed for the external verification. Survival analysis, ROC curves, risk score distribution, survival status, and heatmap of these nine apoptosis-related genes in low- and high-risk groups were displayed in Figure 5, finding similar results as external verification. ROC curves and their associated AUCs for internal verification were also performed and these AUCs also indicated a moderate accuracy, sensitivity and specificity (Table 2).

### Investigation of Independent Prognostic Factors of ccRCC Patients

**FIGURE 6:**
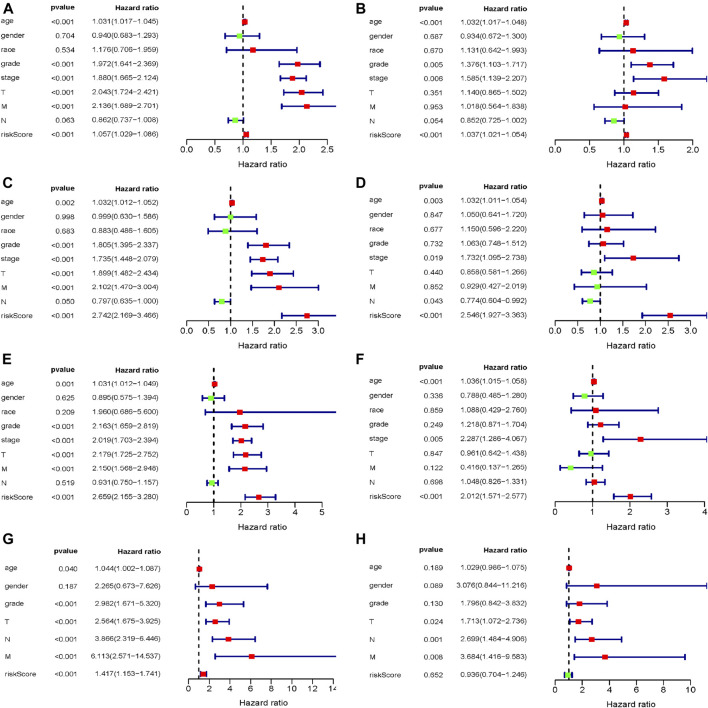
The evaluation of independent prognostic factor; **(A,B)** Univariate and multivariate Cox regression analysis of the whole training dataset (TCGA); **(C,D)** Univariate and multivariate Cox regression analysis of the internal validation dataset 1 (test 1); **(E,F)** Univariate and multivariate Cox regression analysis of the internal validation dataset 2 (test 2); **(G,H)** Univariate and multivariate Cox regression analysis of the external validation dataset (ArrayExpress).

To assess the prognostic significance of different clinical characteristics in ccRCC patients, we performed univariate Cox regression analysis, thus finding that the riskScore entirely could be the prognostic parameters (The whole TCGA dataset: 95%CI 1.029−1.086, *p* < 0.001; The internal validation dataset 1 (test 1): 95%CI 2.169−3.466, *p* < 0.001; The internal validation dataset 2 (test 2): 95%CI 2.155−3.280, *p* < 0.001; The external ArrayExpress dataset: 95%CI 1.153−1.741, *p* < 0.001; respectively) (Figures 6A,C,E, G and Table 3). By the multivariate Cox regression analysis, the nine-genes signature was significantly related with OS in ccRCC patients in the whole TCGA dataset: 95%CI 1.021−1.054, *p* < 0.001; in the internal validation dataset 1 (test 1): 95%CI 1.927−3.363, *p* < 0.001; in the internal validation dataset 2 (test 2): 95%CI 1.571−2.577, *p* < 0.001; except for the ArrayExpress dataset: 95%CI 0.704−1.246; *p* = 0.652 (Figures 6B,D,F,H and Table 3). After calculating the 5 years’ multiROC, the results were as follows: riskScore (AUC = 0.767); age (AUC = 0.601); gender (AUC = 0.484); race (AUC = 0.496); grade (AUC = 0.653); stage (AUC = 0.700); T (AUC = 0.683); M (AUC = 0.631); N (AUC = 0.444). Therein, our established riskScore had the highest 5 years’ AUC, suggesting the best diagnostic performance (Supplementary Figure S2).

**TABLE 3:** Univariate and multivariate Cox regression analysis of external and internal verification datasets for overall survival (OS).

The TCGA dataset								
	**Univariate cox regression analysis**				**Multivariate cox regression analysis**			
**id**	**HR**	**HR.95L**	**HR.95H**	***p* value**	**HR**	**HR.95L**	**HR.95H**	***p* value**
Age	1.030913	1.017301	1.044707	**7.14E-06**	1.032118	1.016820	1.047646	**3.34E-05**
Gender	0.939895	0.682968	1.293475	0.703594	0.934450	0.671839	1.299713	0.687141
Race	1.175718	0.705799	1.958508	0.534112	1.130970	0.641695	1.993303	0.670363
Grade	1.971561	1.640971	2.368751	**4.20E-13**	1.375779	1.102542	1.716731	**0.004741**
Stage	1.880446	1.664529	2.124370	**3.38E-24**	1.585271	1.138915	2.206560	**0.006315**
T	2.043146	1.724210	2.421079	**1.57E-16**	1.140199	0.865365	1.502317	0.351148
M	2.135679	1.688608	2.701115	**2.42E-10**	1.018029	0.563829	1.838118	0.952736
N	0.862088	0.737405	1.007852	0.062627	0.852315	0.724642	1.002482	0.053604
RiskScore	1.057268	1.029085	1.086224	**5.35E-05**	1.037287	1.021209	1.053618	**4.36E-06**
The internal validation dataset 1 (test 1)								
	**Univariate cox regression analysis**				**Multivariate cox regression analysis**			
**id**	**HR**	**HR.95L**	**HR.95H**	***p* value**	**HR**	**HR.95L**	**HR.95H**	***p* value**
Age	1.031655	1.011527	1.052183	**0.001935**	1.032193	1.010596	1.054252	**0.003314**
Gender	0.999353	0.629670	1.586079	0.997809	1.049681	0.640759	1.719571	0.847324
Race	0.882848	0.485694	1.604758	0.682776	1.149877	0.595699	2.219606	0.677270
Grade	1.805395	1.394918	2.336661	**7.16E-06**	1.063436	0.747936	1.512022	0.731959
Stage	1.734982	1.447781	2.079155	**2.41E-09**	1.732016	1.095462	2.738461	**0.018771**
T	1.899001	1.481831	2.433614	**4.03E-07**	0.857855	0.581170	1.266264	0.440278
M	2.101665	1.470228	3.004292	**4.62E-05**	0.928500	0.426908	2.019434	0.851559
N	0.796564	0.634777	0.999587	**0.049584**	0.774358	0.604275	0.992314	**0.043286**
RiskScore	2.741796	2.168650	3.466416	**3.46E-17**	2.546047	1.927273	3.363485	**4.75E-11**
The internal validation dataset 2 (test 2)								
	**Univariate cox regression analysis**				**Multivariate cox regression analysis**			
**id**	**HR**	**HR.95L**	**HR.95H**	***p* value**	**HR**	**HR.95L**	**HR.95H**	***p* value**
Age	1.030515	1.012188	1.049174	**0.001027**	1.036224	1.015147	1.057740	**0.000690**
Gender	0.895400	0.575189	1.393874	0.624636	0.787901	0.484990	1.280002	0.335618
Race	1.959774	0.685844	5.599987	0.209115	1.088336	0.429119	2.760249	0.858511
Grade	2.162630	1.659133	2.818924	**1.17E-08**	1.218212	0.871093	1.703655	0.248714
Stage	2.019196	1.703214	2.393800	**5.83E-16**	2.286627	1.285656	4.066924	**0.004874**
T	2.178898	1.725274	2.751793	**6.19E-11**	0.961049	0.642236	1.438126	0.846811
M	2.150112	1.567994	2.948343	**2.01E-06**	0.415575	0.136574	1.264540	0.121966
N	0.931186	0.749643	1.156694	0.519341	1.048413	0.825956	1.330784	0.697621
RiskScore	2.658673	2.154822	3.280337	**7.44E-20**	2.012308	1.571390	2.576944	**3.00E-08**
The external ArrayExpress dataset								
	**Univariate cox regression analysis**				**Multivariate cox regression analysis**			
**id**	**HR**	**HR.95L**	**HR.95H**	***p* value**	**HR**	**HR.95L**	**HR.95H**	***p* value**
Age	1.043727	1.001971	1.087222	**0.039927**	1.029319	0.985908	1.074642	0.188702
Gender	2.265277	0.672896	7.625960	0.186736	3.076278	0.843743	11.216080	0.088654
Grade	2.981514	1.670809	5.320429	**0.000218**	1.796422	0.842190	3.831833	0.129620
T	2.564016	1.675159	3.924510	**1.45E-05**	1.712645	1.072130	2.735816	**0.024360**
N	3.866228	2.318750	6.446455	**2.17E-07**	2.698628	1.484296	4.906430	**0.001135**
M	6.113103	2.570636	14.537269	**4.20E-05**	3.684199	1.416411	9.582900	**0.007501**
RiskScore	1.416937	1.153316	1.740816	**0.000906**	0.936410	0.703967	1.245604	0.651757

Bold values indicated p < 0.05.

### Construction of a Nomogram Based on our Established Signature and Clinical Characteristics

**FIGURE 7:**
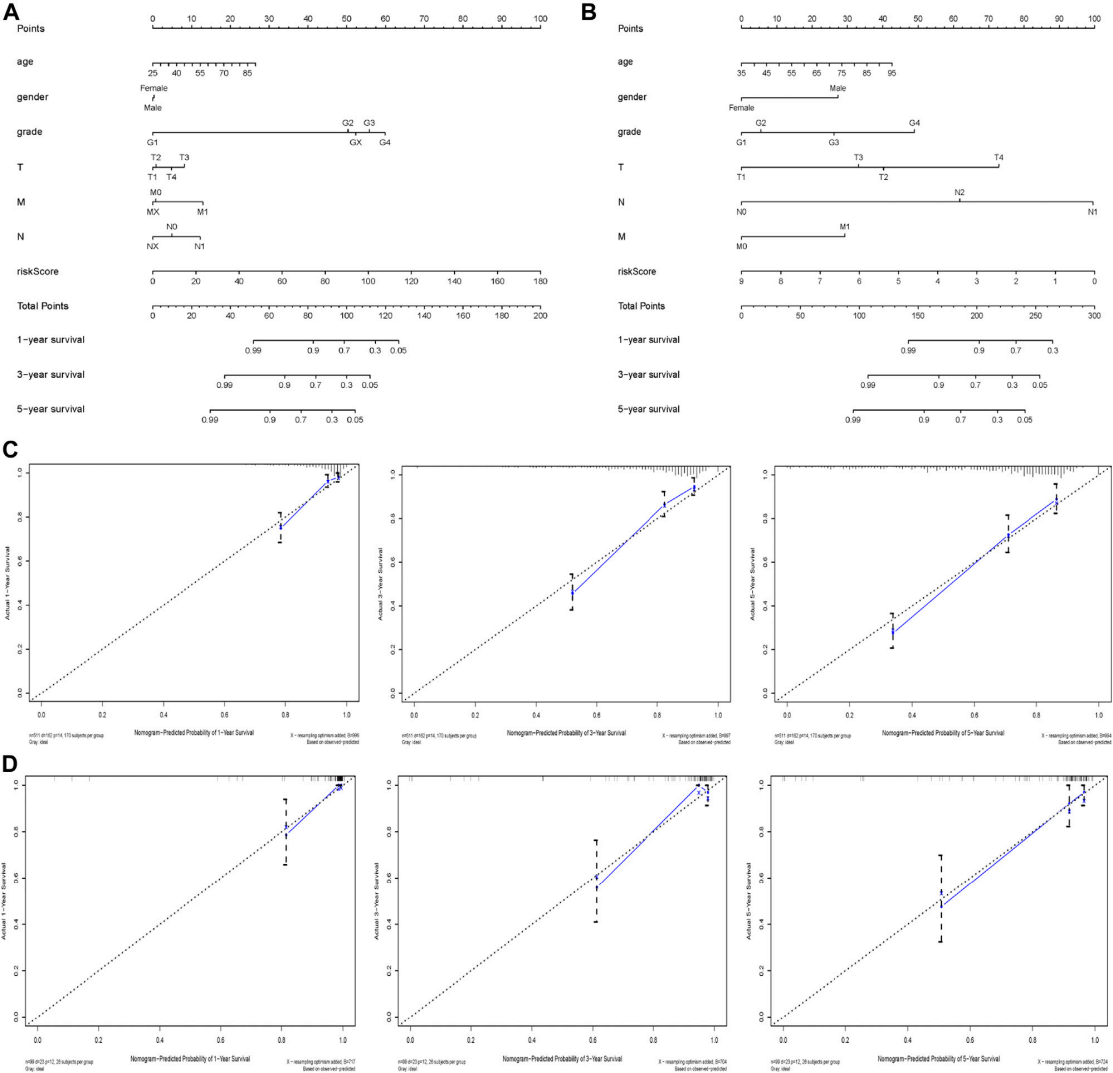
Nomogram and calibration plots based on our established signature and clinical characteristics in both TCGA and ArrayExpress databases; **(A,B)** Nomogram for predicting probabilities of patients with ccRCC with 1-, 3- and 5-year OS in the TCGA and ArrayExpress databases respectively; **(C,D)** The calibration plot of the nomogram for agreement test between 1-, 3- and 5-years OS prediction and real outcome in the TCGA and ArrayExpress databases separately.

In order to help clinicians to develop diagnostic decision-making for ccRCC patients, we combined the nine genes signature and six clinical characteristics (age, gender, grade, T, M and N) to establish a nomogram according to TCGA datasets (Figure 7A). Points were distributed to individual parameters by applying the point scale in the nomogram, and we worked out the total points by summing the points of all factors. We could figure out the survival rates for ccRCC patients at 1, 3, and 5 years, helping the prognostic method for ccRCC patients more quantitative. Its 1-, 3-, 5-year AUC values in the TCGA were 0.846, 0.804, 0.769, with C-index 0.786, thus having good performance in predicting OS (Table 4 and Supplementary Figures S3A–C). Its calibration plots indicated that the predictive values were excellent (Figure 7C). Similarly, we also created another nomogram of the ArrayExpress datasets as the verification (Figure 7B) and its 1-, 3-, 5-years AUC values in the TCGA were 0.925, 0.933, 0.897, with C-index 0.866 (Table 4 and Supplementary Figures S3D–F). Calibration plots of the ArrayExpress dataset showed excellent results too (Figure 7D).

**TABLE 4:** 1-year, 3-years, 5-year ROC and C-index of nomogram in the training dataset (TCGA) and the external validation dataset (ArrayExpress).

	1-year ROC	3-year ROC	5-year ROC	C-index
TCGA cohort	0.846	0.804	0.769	0.786
ArrayExpress cohort	0.925	0.933	0.897	0.866

### Relationships Between RiskScore, These Nine Elected Genes and Clinical Characteristics

To study associations between our established riskScore and clinical characteristics, our results indicated that riskScore were significantly related to grade (*p* = 0.004), tumor stage (*p* = 0.005), T stage (*p* = 0.007) (Supplementary Figure S4). Moreover, clinical correlation analyses between these 9 prognostic apoptosis-related genes, our established riskScore and clinical features such as age, race, gender, grade, stage, T, M, N were also investigated. Therein, *p* values <0.05 indicated a significant correlation (Supplementary Figure S5).

### Prognostic Value of Nine Apoptosis-Related Genes and Clinicopathological Parameters Stratified by our Established riskScore for OS

**FIGURE 8:**
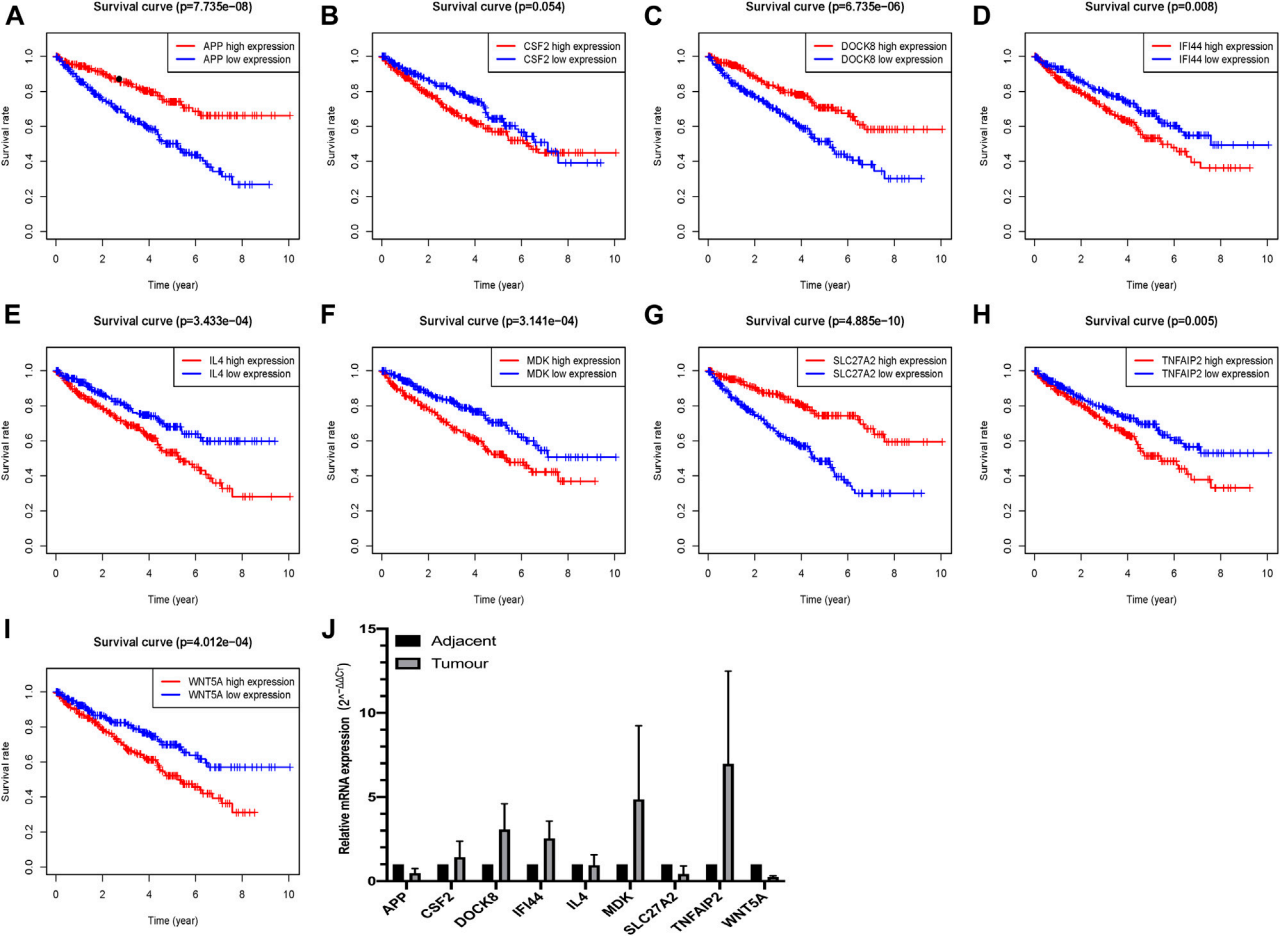
Verification of the expression and prognostic significance of nine critical apoptosis-related genes in ccRCC; **(A)**Survival analysis of APP; **(B)** Survival analysis of CSF2; **(C)** Survival analysis of DOCK8; **(D)** Survival analysis of IFI44; **(E)** Survival analysis of IL4; **(F)** Survival analysis of MDK; **(G)** Survival analysis of SLC27A2; **(H)** Survival analysis of TNFAIP2; **(I)** Survival analysis of WNT5A; **(J)** qRT-PCR verification of these nine critical apoptosis-related genes in ccRCC.

Survival analyses of these nine critical apoptosis-related genes (APP, CSF2, DOCK8, IFI44, IL4, MDK, SLC27A2, TNFAIP2 and WNT5A) were conducted with all *p* < 0.05 (Figures 8A–I), except for survival analysis of CSF2 with *p* value of 0.054, indicating that patients with high expression of APP, DOCK8, SLC27A2 could have a higher chance of better survival performance, while the IFI44, IL4, MDK, TNFAIP2 and WNT5A being the opposite. Moreover, qRT-PCR verified seven apoptosis-related genes (APP, DOCK8, IFI44, MDK, SLC27A2, TNFAIP2, WNT5A) with at least 2 fold changes by means of 2^−ΔΔCt^ method, finding that APP, SLC27A2 and WNT5A had a low expression, while DOCK8, IFI44, MDK, TNFAIP2 had a high expression in ccRCC tumor tissues (Figure 8J).

**FIGURE 9:**
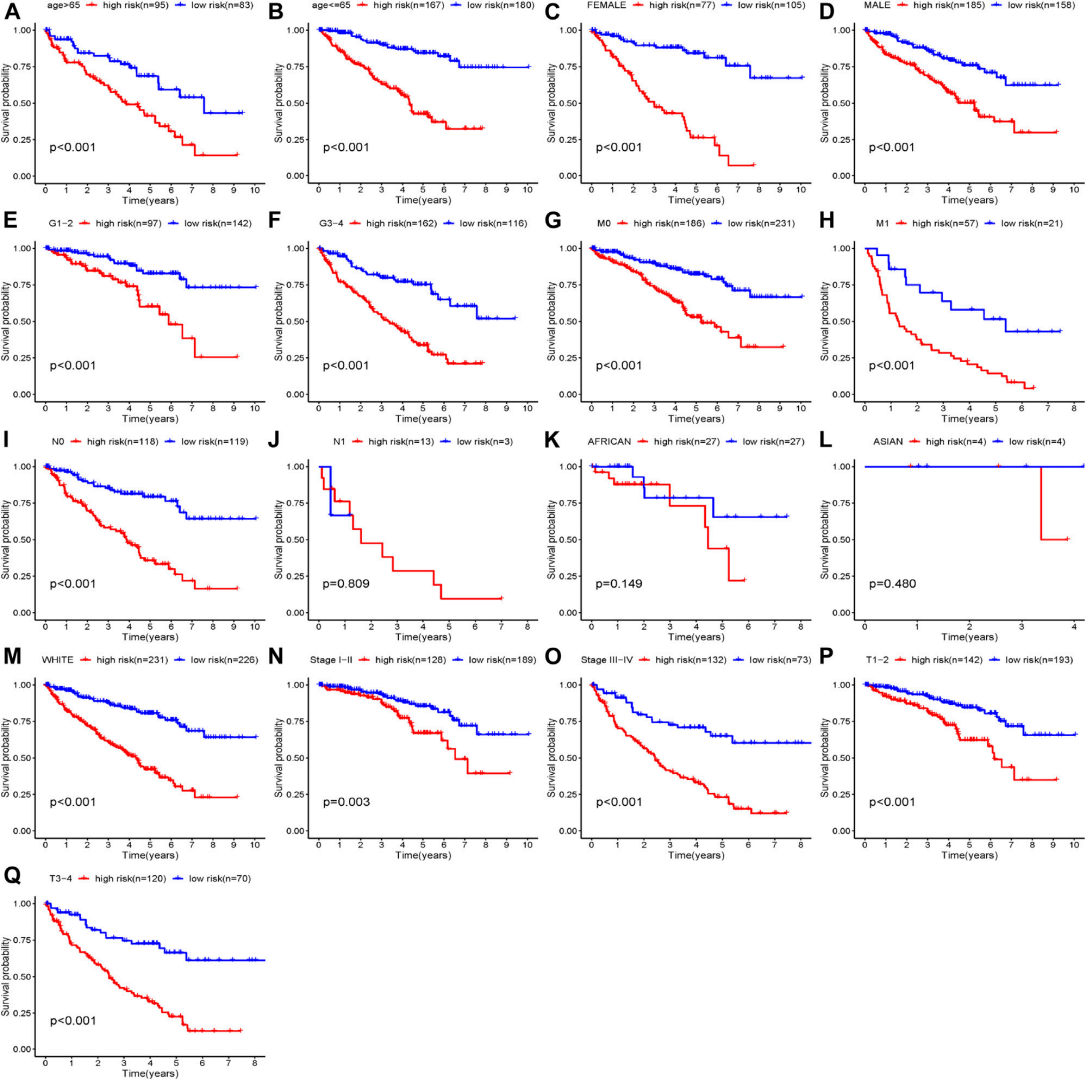
Clinical parameters stratified by our established riskScore for OS; **(A)** Age>65 stratified by riskScore for OS; **(B)** Age<=65 stratified by riskScore for OS; **(C)** Female stratified by riskScore for OS; **(D)** Male stratified by riskScore for OS; **(E)** Grade1-2 stratified by riskScore for OS; **(F)** Grade3-4 stratified by riskScore for OS; **(G)** M0 stratified by riskScore for OS; **(H)** M1 stratified by riskScore for OS; **(I)** N0 stratified by riskScore for OS; **(J)** N1 stratified by riskScore for OS; **(K)** African stratified by riskScore for OS; **(L)** Asian stratified by riskScore for OS; **(M)** White stratified by riskScore for OS; **(N)** Stage I-II stratified by riskScore for OS; **(O)** Stage III-IV stratified by riskScore for OS; **(P)** T1-2 stage stratified by riskScore for OS; **(Q)** T3-4 stage stratified by riskScore for OS.

According to our established riskScore for OS, eight clinical parameters composed of age, gender, grade, stage, race, T, M, N were divided into two subgroups. Our studies indicated that our riskScore worked well in predicting OS in age > 65 (*p* < 0.001), age <=65 (*p* < 0.001), Female (*p* < 0.001), Male (*p* < 0.001), Stage I-II (*p* = 0.002), Stage III-IV (*p* < 0.001), Grade1-2 (*p* < 0.001), Grade3-4 (*p* < 0.001), T1-2 stage (*p* = 0.003), T3-4 stage (*p* < 0.001), N0 (*p* < 0.001), M0 (*p* < 0.001), M1 (*p* < 0.001) and White (*p* < 0.001). Therein, African race, Asian race and N1 had no statistical significance with *p* values > 0.05 (Figure 9).

### Tumor-Infiltrating Immune Cells Between High-and Low-Risk Patients with ccRCC

**FIGURE 10:**
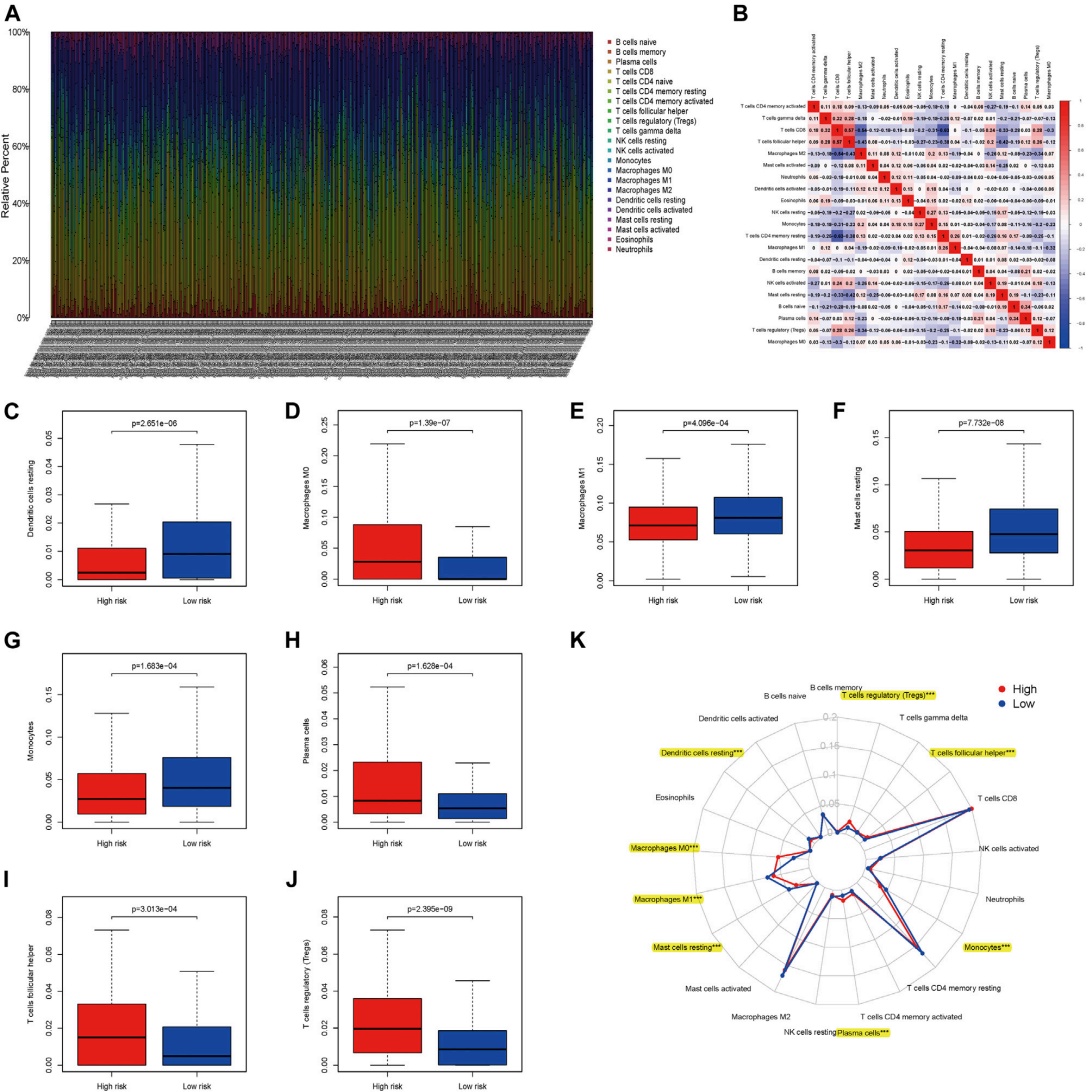
Associations between apoptosis-related genes signature and tumor-infiltrating immune cells (TIICs) in ccRCC; **(A)** The expression of TIICs of all ccRCC samples. **(B)** Correlation matrix of all TIICs proportions; **(C)** Dendritic cells resting between high- and low-risk patients with ccRCC; **(D)** Macrophages M0 between high- and low-risk patients with ccRCC; **(E)** Macrophages M1 between high- and low-risk patients with ccRCC; **(F)** Mast cells resting between high- and low-risk patients with ccRCC; **(G)** Monocytes between high- and low-risk patients with ccRCC; **(H)** Plasma cells between high- and low-risk patients with ccRCC; **(I)** T cells follicular helper between high- and low-risk patients with ccRCC; **(J)** T cells regulatory (Tregs) between high- and low-risk patients with ccRCC; **(K)** The difference of immune cell infiltration abundances in ccRCC subtypes.

As showed in the heatmap, we could observe that the different composition of TIICs in each ccRCC patients (Figure 10A). Also the inner relationship of these TIICs of ccRCC based on the TCGA database was exhibited in the matrix, showing that CD8 T cells was positively correlated with T cells follicular helper, while negatively correlated with M2 macrophages and CD4 T cells memory resting (Figure 10B). Moreover, eight TIICs (Dendritic cells resting, Macrophages M0, Macrophages M1, Mast cells resting, Monocytes, Plasma cells, T cells follicular helper and T cells regulatory) were infiltrated out that represents high correlations between high- and low- risk patients with ccRCC (all *p* < 0.05; Figures 10C–J). As showed in Figure 10K, it detailed the associations of 21 TIICs in high- and low- risk patients with ccRCC. Therein, eight out of these 21 TIICs had significant correlations (all *p* < 0.05).

### Identification of Related Small Molecule Drugs

We applied cMap database to seek out potential small molecule drugs highly correlated to the uploaded differently expressed apoptosis-related genes of ccRCC. Then we obtained the most potential small molecule drugs based on these genes. As showed in Table 5, the small molecule drug named nocodazole was positively related to ccRCC, indicating the potential mechanism of promoting this disease. Furthermore, eight small molecule drugs were negatively correlated with ccRCC containing atractyloside, captopril, coralyne, epitiostanol, clebopride, SR-95531, amiprilose, and harpagoside, indicating the potential repression on the development of this disease.

**TABLE 5:** Results of connectivity map (cMap) analysis.

Rank	cMap name	Mean	n	Enrichment	*p*	Specificity	Percent non-null
1	Atractyloside	−0.486	5	−0.699	0.00539	0.0227	80
2	Captopril	−0.486	5	−0.668	0.00953	0.0318	80
3	Coralyne	−0.479	4	−0.644	0.03847	0.0269	75
4	Epitiostanol	−0.47	4	−0.645	0.03776	0.0617	75
5	Clebopride	−0.462	4	−0.715	0.01337	0.0066	75
6	SR-95531	−0.431	4	−0.629	0.04657	0.0491	75
7	Amiprilose	−0.421	4	−0.634	0.04351	0.0294	75
8	Harpagoside	−0.406	4	−0.85	0.00095	0	75
9	Nocodazole	0.45	6	0.688	0.00238	0.0319	66

## Discussion

Dysregulation of apoptosis-related genes has been reported in a variety of malignant tumors (Huerta et al., 2006; Xu et al., 2009; Cao and Tait, 2018; Yang et al., 2018). However, a few of the apoptosis related genes have studied deeply and reported being associated with both the occurrence of and the inhibition of the progress of renal cancers (Guo et al., 2020; Li et al., 2020). In this study, we combined the RNA-sequencing data and clinical data of ccRCC from TCGA database, thus finding 127 differently-expressed apoptosis-related genes between ccRCC and normal tissue. After the comprehensive investigation to related molecular function and biological action pathway, we constructed and visualized the PPI network of these apoptosis-related genes. We also conducted the LASSO, unadjusted and adjusted Cox regression analyses to identify the hub candidate genes. Moreover, we constructed the risk model to conduct the prediction of prognosis based on nine hub apoptosis-related genes. The evaluation of the model was performed with survival analyses and ROC analyses of these nine hub apoptosis-related genes in both the TCGA and ArrayExpress databases. These findings may make a contribution to the development of accurate and sensitive biomarkers for the diagnosis and prognosis to the ccRCC patients.

GO enrichment analysis suggested that these genes are mainly enriched in regulation of lymphocyte activation, positive regulation of leukocyte activation, positive regulation of cell activation, neuron death, neuron apoptotic process, immunoglobulin receptor binding, antigen binding, scavenger receptor activity, cargo receptor activity, receptor ligand activity, immunoglobulin complex, blood microparticle, and membrane raft. The role of apoptosis in human diseases, especially cancer, has been increasingly recognized in many studies (Yu et al., 2015; Pai et al., 2017; Fathi et al., 2018). As for apoptosis-related genes, they often played critical roles in tumorigenesis and involved in the control of cancer progression (Fulda, 2015; Cao and Tait, 2018). Several apoptosis-related genes, had been reported to affect renal cell activity and cell proliferation (Woo et al., 2018). The degree of miR-182 was markedly reduced in renal cancer tissue, while mTOR was upregulated (Fu et al., 2018). In regards to ccRCC, down-regulated XIAP expression could increase the apoptosis-related sensitivity of RCC cells (Yang et al., 2018). In prostate cancer, FOXQ1 regulated the expression of BCL11A/MDM2 to promote cell proliferation (Zhang et al., 2016).

Additionally, results of KEGG pathway analysis indicated that apoptosis-related genes were mainly enriched in Cytokine-cytokine receptor interaction, Allograft rejection, Epstein-Barr virus infection, Primary immunodeficiency, PI3K-Akt signaling pathway and Th1 and Th2 cell differentiation. As previously reported, apoptotic factors expressed by apoptotic-related genes can combine with cytochrome C released into the cytoplasm to form a polymer within the abundance of dATP, and induce apoptosis by binding the relevant gene caspase-9 to form apoptotic corpuscle (Shi, 2002). Fas, a member of the tumor necrosis factor receptor superfamily, expressed by related factors is a transmembrane protein, can initiate apoptosis signal transduction and induce apoptosis when combined with FasL (Curtin and Cotter, 2003). Therefore, the development of pathways exploration worked well, and other biological mechanisms needed to be further studied. Further, we constructed the PPI network and applied the MODE plug-in to identify three vital modules of these differently-expressed apoptosis-related genes.

By means of LASSO, unadjusted and adjusted Cox regression analyses, we acquired nine hub genes (SLC27A2, TNFAIP2, IFI44, CSF2, IL4, MDK, DOCK, WNT5A, APP). Previous studies indicated that these genes were closely related to the occurrence and progress of carcinoma (Hoon et al., 1991; Cho and Kim, 2017; Chen et al., 2018). For example, TNFα-induced protein 2 (TNFAIP2) played a functional role in cell proliferation, angiogenesis, migration and invasion (Jia et al., 2018). Furthermore, interferon-induced protein 44-like (IFI44L) gene served as a type of interferon-stimulated gene (ISG), and we found that consumption of IFI44L activated Met/Src signaling pathway to promote migration, invasion and metastasis (Huang et al., 2018). Additionally, it had been shown that adverse clinical outcomes were caused through overexpression of CSF2 in urothelial carcinoma and epithelial carcinoma of the bladder (Lee et al., 2016). Moreover, Midkine had differently expression at high levels in malignant tumors and acted as a critical substance to promote the growth and metastasis of cancers (Filippou et al., 2020). As for Interleukin-4 (IL4), Hoon, D. S. et al. demonstrated that IL4 alone could modulate the expression of tumor-associated of renal cell carcinomas (Hoon et al., 1991).

We then established the prognostic model based on the nine hub genes. According to the model, the curves based on the two groups indicated that the patients in the low-risk subgroup were with high OS in the TCGA cohort, the external validation dataset (ArrayExpress) and the internal validation dataset (dataset 1; dataset 2). The time-dependent ROC curve and the AUCs of the model demonstrated that these selected genes had a moderate performance when predicting OS at 1-, 3- and 5-year. In regard to the results of univariate and multivariate Cox regression analysis, the riskScore could serve as an independent factor of OS. Among the validation of the four cohorts, the *p* values of the riskScores were all <0.05, except for the ArrayExpress dataset which may be due to an inadequate sample. Therefore, the high sensitivity and accuracy of the nine genes signature in predicting OS for ccRCC patients was acknowledged. Additionally, we constructed a nomogram consisting of several clinical factors and the risk score to predict the 1-, 3- and 5-year OS, finding satisfactory outcomes in the external and internal datasets.

We also conducted further validation of these nine apoptosis-related genes in renal tissues by a series of qRT-PCR, thus offering strong evidence to the above conclusion. Furthermore, we explored its association with immune infiltration and applied cMap database to seek out potential small molecule drugs highly correlated to the differently expressed apoptosis-related genes in ccRCC. Our analysis is undoubtedly helpful, but application in future research is warranted.

The strength of this article was that it was the first time we explored the roles of apoptosis-related genes in ccRCC. Moreover, our prognostic model was not only based on the TCGA database, but also verified by the data from the external database ArrayExpress and two internal validation datasets, making the results more accurate. Further, our results remained stable internally and externally, making the verification more sufficient and the experimental results more persuasive. However, this study still had some limitations. First, our study was retrospective in nature, requiring the use of prospective studies to validate the findings. Second, when detecting the independent factors, we found that *p*-value of ArrayExpress database = 0.652 (*p* > 0.05) in the multivariate Cox proportional hazards regression, potentially attributing to the inadequacy of the samples from the ArrayExpress datasets. Finally, the establishment of our prognostic model and the evaluation of independent factors mainly focused on the survival time. The selection of clinical treatment plans, treatment information (precise treatment sequence, with or without co-morbidities, etc), a prolonged course of disease, as well as the early and late quality of life was not involved.

## Conclusion

By means of external and internal validation, our study successfully constructed a prognostic model containing nine hub apoptosis-related genes (SLC27A2, TNFAIP2, IFI44, CSF2, IL4, MDK, DOCK, WNT5A, APP). Moreover, our established signature could serve as a tool to help clinicians predict patients’ OS, allowing for a more standardized prognostic assessment. Future prospective studies are required to validate the efficiency and accuracy of our work to improve the diagnosis and management of ccRCC.

## Data Availability

The datasets presented in this study can be found in online repositories. The names of the repository/repositories and accession number(s) can be found in the article. R and Perl scripts involved in this article were displayed in Supplementary Material.
